# An Exploratory Indirect Association Analysis of Dietary Supplement Use and Metabolic Risk in Korean Adolescents: A Cross-Sectional Study

**DOI:** 10.3390/foods15142554

**Published:** 2026-07-20

**Authors:** Jeoungmi Kim, Vasuki Rajaguru

**Affiliations:** 1 Department of Nursing, Kaya University, Gimhae 50382, Republic of Korea; jeoung66@kaya.ac.kr; 2 Department of Healthcare Management, Graduate School of Public Health, Yonsei University, Seoul 03722, Republic of Korea

**Keywords:** dietary supplement use, diet quality, body mass index, metabolic risk clustering, adolescent health, nutrition

## Abstract

Aims: Dietary supplement use has increased among adolescents, but its association with metabolic health remains unclear. This study examined the relationship between dietary supplement use, diet quality, obesity, and metabolic risk among Korean adolescents. Methods: This cross-sectional study used nationally representative KNHANES 2022–2024 data. A total of 1550 adolescents aged 10–18 years were included. Dietary supplement use during the previous year was assessed by questionnaire. Diet quality was evaluated using diet quality score based on selected dietary guideline-related components. Survey-weighted logistic and linear regression analyses were conducted after adjustment for demographic and dietary covariates. An exploratory indirect association analysis was performed to examine whether diet quality was statistically associated with the relationship between supplement use and metabolic risk clustering. Results: Overall, 43.1% of adolescents reported dietary supplement use. Supplement users had significantly better diet quality scores than non-users (β = 0.30, *p* < 0.001). Dietary supplement use was significantly associated with BMI (*p* = 0.011) and waist circumference (*p* = 0.010), but not with metabolic risk clustering (OR = 0.91, 95% CI: 0.67–1.24), fasting glucose, triglycerides, or HDL-cholesterol levels. Obesity was strongly associated with metabolic risk clustering regardless of supplement use. Compared with non-obese non-users, obese non-users (OR = 5.31, 95% CI: 3.22–8.78) and obese supplement users (OR = 3.68, 95% CI: 2.07–6.53) had significantly higher odds of metabolic risk clustering. Exploratory indirect association analysis showed that diet quality did not significantly mediate the association between dietary supplement use. Conclusions: Dietary supplement use was associated with better diet quality and favorable anthropometric indicators, but not with metabolic risk clustering or biochemical metabolic markers. These findings suggest that supplement use may reflect broader health-related behaviors rather than direct metabolic protection among Korean adolescents.

## 1. Introduction

Adolescence is a critical developmental period characterized by rapid physical growth, hormonal changes, and the establishment of long-term health behaviors [1,2]. Nutritional status during adolescence plays an important role in growth, metabolic regulation, and future risk of chronic diseases such as obesity, diabetes, hypertension, and cardiovascular disease [3,4]. In recent years, unhealthy dietary behaviors among adolescents, including increased consumption of processed foods, excessive sodium intake, meal skipping, and reduced fruit and vegetable consumption, have become major public health concerns globally and in Korea [5]. These dietary transitions have contributed to increasing rates of adolescent obesity and metabolic abnormalities, highlighting the importance of promoting healthy eating behaviors during early stages.

Dietary supplement use has also increased substantially among children and adolescents worldwide [6]. Previous studies have reported that adolescents often consume dietary supplements to improve general health, compensate for perceived nutritional inadequacies, enhance immunity, or support growth and physical performance [7,8]. In Korea, dietary supplement use among adolescents has become increasingly common due to growing health awareness and parental interest in nutritional support [9,10]. However, evidence regarding the health benefits of dietary supplement use among adolescents remains inconsistent. While some studies suggest that supplement users tend to exhibit healthier lifestyles and better nutritional profiles, others report limited evidence for direct metabolic or clinical benefits [11,12].

Diet quality is an important determinant of metabolic health during adolescence. Healthy dietary patterns characterized by balanced nutrient intake, regular meals, and higher consumption of fruits and vegetables have been associated with lower obesity risk and improved cardiometabolic outcomes [13,14]. Conversely, unhealthy dietary patterns high in sodium, sugar, and processed foods are associated with obesity, dyslipidemia, insulin resistance, and metabolic syndrome risk factors among adolescents [15,16]. Despite increasing interest in dietary supplement use, relatively few studies have explored whether diet quality mediates the relationship between supplement use and metabolic health, particularly among Korean adolescents [9,17,18].

Understanding these relationships is especially important in Korea, where rapid lifestyle changes and westernized dietary patterns have altered adolescent nutritional behaviors over recent decades. National reports have shown increasing obesity prevalence and dietary imbalance among Korean youth, emphasizing the need for effective nutrition-related public health interventions [19,20].

Despite the increasing use of dietary supplements among Korean adolescents, evidence regarding their relationship with overall diet quality and cardiometabolic health remains limited and inconsistent. Previous Korean studies have primarily described the prevalence, determinants, or nutritional characteristics of dietary supplement use, whereas few have simultaneously examined dietary supplement use, diet quality, obesity, and metabolic risk using nationally representative data [21,22,23]. Moreover, although healthier dietary patterns have been associated with more favorable metabolic outcomes in adolescents, it remains unclear whether diet quality is associated with the relationship between dietary supplement use and metabolic risk in this population [9]. Because dietary supplement users often exhibit healthier lifestyle behaviors than non-users, distinguishing the independent contribution of supplement use from overall diet quality is an important public health question [24,25].

Therefore, the present study aimed to examine the associations between dietary supplement use, diet quality, obesity, and metabolic risk clustering among Korean adolescents using nationally representative KNHANES 2022–2024 data. In addition, we conducted an exploratory indirect association analysis to investigate whether diet quality was associated with the relationship between dietary supplement use and metabolic risk clustering. Given the cross-sectional design, these analyses were intended to explore potential associations rather than infer causal pathways or mediation.

## 2. Materials and Methods

### 2.1. Study Design and Data Source

This cross-sectional study used data from the Korea National Health and Nutrition Examination Survey (KNHANES) 2022–2024 conducted by the Korea Disease Control and Prevention Agency [26]. KNHANES is a nationally representative survey designed to assess the health and nutritional status of the Korean population using a stratified, multistage probability sampling design. The survey includes health interviews, health examinations, and nutrition assessments conducted by trained personnel.

### 2.2. Study Population

The initial pooled KNHANES 2022–2024 dataset included 20,191 participants. Adolescents aged ≤18 years were selected for the present study. Participants with missing information on dietary supplement use, metabolic biomarkers, dietary intake variables, or covariates were excluded from the final analysis. A total of 1550 Korean adolescents were included in the descriptive analyses, while regression analyses were conducted using participants with complete data for the corresponding models (Supplementary Figure S1).

### 2.3. Outcome Variables

The primary outcome of this study was metabolic risk clustering among Korean adolescents. Metabolic risk clustering was defined as the presence of two or more abnormal metabolic components, including general obesity (BMI ≥ 95th percentile for age and sex or ≥25.0 kg/m^2^), abdominal obesity (waist circumference ≥ 90th percentile for age and sex), elevated fasting glucose (≥100 mg/dL), hypertriglyceridemia (≥150 mg/dL), low HDL-cholesterol (<40 mg/dL), and elevated blood pressure (SBP ≥ 130 mmHg and/or DBP ≥ 85 mmHg) [10], obtained from the KNHANES health examination survey. Detailed diagnostic criteria are presented in Supplementary Tables S1 and S2. The primary exposure variable was dietary supplement use during the previous year, categorized as supplement users and non-users based on self-reported questionnaire responses. Secondary outcomes included diet quality score and BMI status. Diet quality was assessed using a modified Korean dietary guideline-based score that included energy intake adequacy, carbohydrate intake proportion, regular meal consumption, vegetable intake frequency, sodium intake, and alcohol-related behavior. The total score ranged from 0 to 6 and was further categorized into poor (0–2 points), moderate (3–4 points), and good (5–6 points) diet quality groups [27] (Supplementary Table S1). And only a small proportion of participants met the criteria for the highest diet quality category, the continuous diet quality score was used in all regression and exploratory indirect association analyses to preserve variability and improve statistical power. BMI categories were classified as underweight, normal weight, overweight, and obese according to BMI levels (Supplementary Table S3).

### 2.4. Covariates

Covariates included sex (male/female), age group (10–12, 13–15, and 16–18 years), household income level (low, lower-middle, upper-middle, and high), and residential area (urban/rural). Additional dietary covariates included total energy intake, protein intake, carbohydrate intake, sodium intake, and carbohydrate percentage derived from nutrition survey data.

### 2.5. Statistical Analysis

All statistical analyses were performed using SAS 9.4 V while accounting for the complex sampling design of KNHANES, including stratification, clustering, and survey weights. Health examination weights, stratification variables, and primary sampling units were applied to obtain nationally representative estimates for Korean adolescents. First, Baseline characteristics were summarized according to dietary supplement use status. Categorical variables were presented as weighted frequencies and percentages, whereas continuous variables were expressed as weighted means and standard errors (SE). Second, differences between supplement users and non-users were assessed using Rao–Scott chi-square tests and survey-weighted mean comparisons. Third, Survey-weighted logistic regression analyses were conducted to examine the association between dietary supplement use and metabolic risk clustering. Odds ratios (ORs) and 95% confidence intervals (CIs) were estimated using two models. Model 1 adjusted for demographic factors, including age group, sex, household income, residential area, and survey year. Model 2 additionally adjusted for dietary intake variables, including total energy intake, sodium intake, and carbohydrate percentage. Sex-stratified analyses were also performed separately for male and female adolescents. Fourth, Survey-weighted linear regression analyses were used to evaluate the associations between dietary supplement use, diet quality score, and BMI. Finally, an exploratory indirect association analysis was conducted to examine whether diet quality was statistically associated with the relationship between dietary supplement use and metabolic risk clustering. Because the study was cross-sectional, these analyses were exploratory and were not interpreted as evidence of causal mediation or temporal pathways. Statistical significance was defined as a two-sided *p* < 0.05, and all analyses were performed using SAS version 9.4 (SAS Institute Inc., Cary, NC, USA).

## 3. Results

### 3.1. General Characteristics of the Participants

Table 1 presents the baseline characteristics of Korean adolescents according to dietary supplement use. Among 1550 participants, 43.1% reported dietary supplement use during the past year. Significant differences were observed for age group, household income, BMI category, and diet quality. Supplement users were more likely to be aged 10–12 years, belong to upper-middle income households, and have better diet quality profiles compared with non-users (all *p* < 0.05). In contrast, overweight prevalence was higher among non-users. No significant differences were found for sex, residence, or metabolic risk clustering between the two groups.

The clinical and nutritional characteristics of Korean adolescents. The mean BMI was 21.17 kg/m^2^, and the average waist circumference was 71.52 cm. Mean fasting glucose, triglyceride, and HDL-cholesterol levels were 91.49 mg/dL, 88.10 mg/dL, and 56.83 mg/dL, respectively. Adolescents consumed an average of 1892.85 kcal/day, with mean protein and carbohydrate intakes of 72.99 g/day and 263.68 g/day, respectively. The average sodium intake was relatively high at 2862.72 mg/day (Supplementary Table S4).

### 3.2. Multivariate Logistic Regression Model Analysis for Metabolic Risk Clustering Among Korean Adolescents

Table 2 exhibits that dietary supplement use was not significantly associated with metabolic risk clustering in either Model 1 (OR = 0.83, 95% CI: 0.61–1.12, *p* = 0.211) or Model 2 after additional adjustment for dietary variables (OR = 0.91, 95% CI: 0.67–1.24, *p* = 0.546). Adolescents aged 13–15 years had significantly lower odds of metabolic risk clustering compared with those aged 10–12 years in both Model 1 (OR = 0.69, 95% CI: 0.50–0.96) and Model 2 (OR = 0.66, 95% CI: 0.47–0.92). Female adolescents also showed consistently lower odds of metabolic risk clustering than males in Model 1 (OR = 0.54, 95% CI: 0.40–0.74, *p* < 0.001) and Model 2 (OR = 0.61, 95% CI: 0.44–0.85, *p* = 0.003). Household income, residence, survey year, total energy intake, sodium intake, and carbohydrate percentage were not significantly associated with metabolic risk clustering.

The distributions of selected dietary and metabolic clustering indicators according to dietary supplement use (Supplementary Figure S2). Compared with non-users, supplement users had a significantly higher diet quality score (β = 0.30, 95% CI: 0.17–0.43; *p* < 0.001). However, no significant differences were observed in fasting glucose (*p* = 0.330), triglycerides (*p* = 0.744), or HDL-cholesterol (*p* = 0.834). Total energy intake, sodium intake, and carbohydrate energy percentage also showed largely overlapping distributions between the two groups, indicating similar overall nutrient intake patterns despite the modestly higher diet quality among supplement users.

### 3.3. Exploratory Indirect Association Analysis

**Figure 1:**
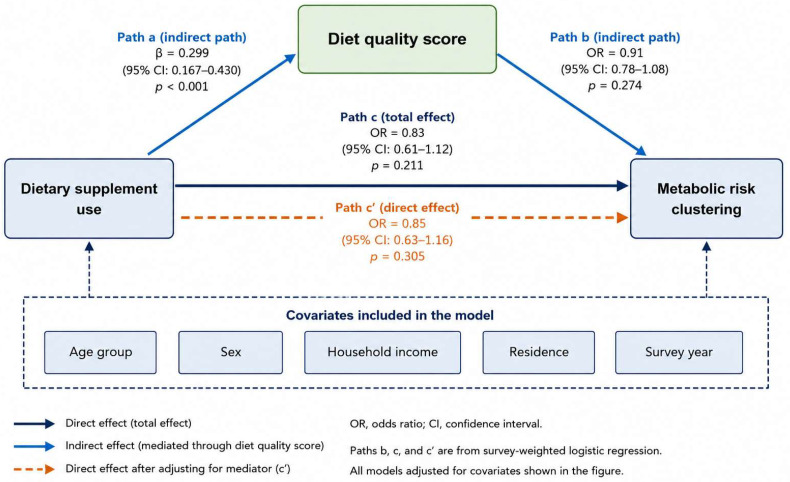
An exploratory indirect association analysis pathway models of dietary supplement use, diet quality, and metabolic risk among Korean adolescents.

Figure 1 presents the exploratory indirect association pathway between dietary supplement use, diet quality score, and metabolic risk clustering among Korean adolescents. Dietary supplement use was significantly associated with higher diet quality scores (Path a: β = 0.299, *p* < 0.001), whereas diet quality score was not significantly associated with metabolic risk clustering (Path b: OR = 0.91, *p* = 0.274). Supplement use also showed no significant association with metabolic risk clustering in either the total effect model (Path c: OR = 0.83, *p* = 0.211) or the direct effect model adjusted for diet quality score (Path c′: OR = 0.85, *p* = 0.305).

### 3.4. Sex Stratified Association Between Metabolic Risk Clustering Among Korean Adolescents

Table 3 presents the sex-stratified survey-weighted logistic regression analyses for metabolic risk clustering. Among male adolescents, dietary supplement use was associated with lower odds of metabolic risk clustering than non-use, although the association did not reach statistical significance (OR = 0.72, 95% CI: 0.49–1.05; *p* = 0.084). No significant associations were observed for age group, household income, residence, or survey year. Among female adolescents, dietary supplement use was not significantly associated with metabolic risk clustering (OR = 1.10, 95% CI: 0.69–1.75; *p* = 0.700). Compared with those aged 10–12 years, female adolescents aged 13–15 years (OR = 0.37, 95% CI: 0.21–0.65; *p* < 0.001) and 16–18 years (OR = 0.53, 95% CI: 0.30–0.91; *p* = 0.022) had significantly lower odds of metabolic risk clustering.

### 3.5. Subgroup Analysis

#### 3.5.1. Metabolic Components According to Dietary Supplement Use

Survey-weighted linear regression analyses showed that dietary supplement use was significantly associated with lower BMI (β = −0.69 kg/m^2^, 95% CI: −1.21 to −0.18, *p* = 0.009) and smaller waist circumference (β = −1.71 cm, 95% CI: −3.02 to −0.41, *p* = 0.010). However, dietary supplement use was not significantly associated with fasting glucose, triglycerides, HDL-cholesterol, systolic blood pressure, or diastolic blood pressure after adjustment for demographic covariates. These findings suggest that supplement use was associated with more favorable anthropometric measures but not with metabolic biomarkers or blood pressure (Supplementary Table S5).

#### 3.5.2. Metabolic Risk Clustering and Supplement Use According to Obesity Status and Interaction Between Sex and Supplement Use

Obesity status was strongly associated with metabolic risk clustering among Korean adolescents. Compared with obese adolescents, non-obese adolescents had significantly lower odds of metabolic risk clustering (aOR = 0.21, 95% CI: 0.14–0.31, *p* < 0.001). Adolescents aged 13–15 years and 16–18 years showed lower odds of metabolic risk clustering than those aged 10–12 years, and female adolescents had lower odds than males. Household income, residence, and survey year were not significantly associated with metabolic risk clustering (Supplementary Table S6). In addition, the interaction between dietary supplement use and sex was not statistically significant (F = 2.82, *p* = 0.094); therefore, the sex-stratified findings should be interpreted as exploratory rather than evidence of effect modification (Supplementary Table S7).

## 4. Discussion

The present study examined the association between dietary supplement use, diet quality, and metabolic risk clustering among Korean adolescents using nationally representative KNHANES 2022–2024 data. The findings demonstrated that dietary supplement users had significantly better diet quality profiles than non-users; however, dietary supplement use was not significantly associated with metabolic risk clustering after adjustment for demographic and dietary factors. In addition, diet quality did not mediate the relationship between supplement use and metabolic risk clustering.

A major finding of this study was that dietary supplement users exhibited healthier dietary behaviors and higher diet quality scores than non-users. Supplement users were less likely to have poor diet quality and more likely to have moderate diet quality, suggesting that dietary supplement use may reflect greater health consciousness among adolescents. These findings are consistent with previous Korean studies reporting that greater adherence to dietary guidelines was associated with improved diet quality and a lower risk of obesity among children and adolescents [28]. Similarly, studies using KNHANES data have shown that healthier eating behaviors among Korean adolescents are associated with improved nutritional status and healthier lifestyle patterns [7,29]. Previous Korean research also reported that dietary supplement use was more common among adolescents with regular meal consumption, healthier dietary practices, and higher household income levels [9,11,12]. The current findings therefore support the idea that dietary supplement use among Korean adolescents is more likely part of an overall health-oriented lifestyle rather than a compensatory behavior for poor dietary habits.

Despite the positive association between supplement use and diet quality, no significant association was observed between dietary supplement use and metabolic risk clustering. This finding remained stable after further adjustment for energy intake, sodium intake, and carbohydrate percentage. Similar findings have been reported in adolescent nutrition studies where healthier dietary patterns were associated with improved nutritional behaviors but showed inconsistent associations with metabolic syndrome components during adolescence [15,30,31,32,33]. One possible explanation is that adolescents generally have a lower prevalence of cardiometabolic abnormalities compared with adults, making it more difficult to detect measurable metabolic differences at younger ages [13]. In the present study, average fasting glucose, triglyceride, and HDL-cholesterol levels were largely within normal ranges, indicating an overall metabolically healthy population.

BMI was included as one component of metabolic risk clustering; the association between obesity and the composite outcome may partly reflect mathematical overlap [34]. Therefore, this finding should be interpreted cautiously [10,31]. To address this potential circularity, we performed a sensitivity analysis excluding BMI from the clustering definition, and the results remained materially unchanged. This supports the robustness of our findings and suggests that the observed associations were not driven solely by the inclusion of BMI in the composite outcome [35].

Interestingly, dietary supplement use was significantly associated with BMI category, although no direct relationship with metabolic risk clustering was identified. Supplement users had higher proportions of underweight adolescents, whereas overweight prevalence was greater among non-users [36]. Previous Korean studies have similarly reported associations between dietary quality, obesity status, and adolescent health behaviors [3,7]. A study found that Korean children with greater adherence to dietary guidelines had significantly lower obesity risk [37]. In addition, recent Korean studies reported that unhealthy dietary behaviors, meal skipping, and sedentary lifestyles were associated with increased obesity risk among adolescents [17,18,29]. These findings suggest that dietary supplement use may be more closely related to body weight management and dietary awareness than to broader metabolic abnormalities.

The exploratory indirect association analysis further showed that diet quality did not significantly mediate the relationship between dietary supplement use and metabolic risk clustering. Although dietary supplement use was positively associated with diet quality score, diet quality itself was not significantly associated with metabolic risk clustering [8,38]. Consequently, neither the total effect nor the direct effect of supplement use on metabolic risk clustering was statistically significant [1,13,25]. This suggests that the healthier dietary behaviors observed among supplement users may not yet translate into measurable metabolic benefits during adolescence [33]. Since metabolic disorders often develop gradually over time, longer-term longitudinal studies may be needed to identify potential cumulative effects of healthy dietary behaviors and supplement use on cardiometabolic health [32]. Because the temporal sequence among dietary supplement use, diet quality, and metabolic risk could not be established, the exploratory indirect association analysis should not be interpreted as evidence of mediation or causality but rather as an assessment of potential relationships among these variables [24,39]. Although dietary supplement users had significantly higher diet quality scores than non-users, the adjusted difference was only 0.30 points on a 0–6 scale. This modest effect size suggests limited clinical relevance and does not necessarily reflect a substantial improvement in overall dietary quality.

Sex-stratified analyses also revealed different patterns between males and females. Among male adolescents, supplement users tended to have lower odds of metabolic risk clustering, although the association was not statistically significant. In contrast, no association was observed among females [38]. Female adolescents overall demonstrated lower odds of metabolic risk clustering than males [33]. These findings are partly consistent with previous evidence indicating sex-related differences in obesity, dietary patterns, and cardiometabolic health among Korean adolescents [10,40]. Biological maturation, hormonal factors, and behavioral differences may contribute to these sex-specific metabolic patterns. Although sex-stratified analyses suggested some differences in the magnitude of the associations, the formal interaction test was not statistically significant. This indicates that the relationship between dietary supplement use and metabolic risk clustering was generally consistent across sexes, and the observed subgroup differences may reflect random variation rather than true biological effect modification.

The findings of this study are particularly important in the Korean public health context, where increasing consumption of processed foods, meal skipping, and dietary imbalance among adolescents have become growing concerns. Recent Korean studies have reported declining fruit, vegetables, and breakfast consumption together with increasing obesity prevalence among adolescents. Korean child welfare and nutrition policies have increasingly emphasized balanced dietary intake, nutrition education, and healthy lifestyle promotion to prevent childhood obesity and future chronic diseases. The current findings suggest that dietary supplement use alone may not provide metabolic advantages during adolescence, highlighting the importance of comprehensive dietary and lifestyle interventions rather than reliance on supplements. Overall, this study suggests that dietary supplement use among Korean adolescents is associated with healthier dietary behaviors and better diet quality, but not with reduced metabolic risk clustering. These findings indicate that supplement use may reflect broader health-conscious lifestyle behaviors rather than direct metabolic protection during adolescence.

### 4.1. Limitations

This study has several limitations. First, its cross-sectional design precludes establishing temporal or causal relationships among dietary supplement use, diet quality, obesity, and metabolic risk. Accordingly, the exploratory indirect association analysis should not be interpreted as evidence of mediation or causality. Second, dietary intake, dietary supplement use, and lifestyle behaviors were self-reported and may be subject to recall and reporting bias. Third, the KNHANES dataset did not provide detailed information on supplement type, composition, dosage, frequency, or duration of use, preventing evaluation of specific supplement effects. Fourth, although multiple demographic factors were adjusted for, residual confounding from unmeasured variables, such as physical activity, pubertal status, parental education, and other health-related behaviors, cannot be excluded. Fifth, because BMI was included as one component of the metabolic risk clustering definition, there is potential for mathematical overlap; however, sensitivity analyses excluding BMI yielded consistent findings. Finally, the relatively low prevalence of metabolic abnormalities and the limited variability in diet quality among Korean adolescents may have reduced the statistical power to detect modest associations. Finally, the inverse associations of dietary supplement use with BMI and waist circumference should be interpreted cautiously. Supplement users may differ from non-users in health awareness, parental support, physical activity, socioeconomic circumstances, and other behaviors that were not fully captured, creating a potential healthy-user bias [41] (Shrank et al., 2011). Moreover, the cross-sectional design does not establish whether supplement use preceded lower adiposity; adolescents or parents concerned about body weight may initiate supplement use, making reverse causality equally plausible. Although the models adjusted for key demographic factors, unmeasured or incompletely measured characteristics may still produce residual confounding, which can alter the magnitude or even the direction of associations in observational studies [42,43]. Therefore, the findings should not be interpreted as evidence that dietary supplements reduce BMI or waist circumference.

### 4.2. Implications

The findings of this study have important implications for adolescent nutrition and public health policies in Korea. Although dietary supplement use was associated with better diet quality and healthier dietary behaviors, it was not linked to lower metabolic risk clustering among Korean adolescents. These results suggest that dietary supplements alone may not provide measurable metabolic benefits during adolescence and should not replace balanced dietary practices and healthy lifestyle behaviors. Public health strategies should therefore focus on improving overall diet quality, promoting regular meal patterns, reducing excessive sodium intake, and encouraging physical activity rather than relying primarily on supplement consumption. In the Korean context, where childhood obesity and dietary imbalance are increasing concerns, school- and community-based nutrition education programs may help adolescents develop sustainable healthy eating habits and long-term metabolic health.

## 5. Conclusions

In this nationally representative study of Korean adolescents, dietary supplement users generally had better diet quality than non-users. However, dietary supplement use was not independently associated with metabolic risk clustering after accounting for demographic and lifestyle-related factors, and diet quality was not associated with metabolic risk clustering. Female adolescents and older age groups consistently showed lower odds of metabolic risk clustering than males and younger adolescents. These findings suggest that dietary supplement use may reflect a broader pattern of health-conscious behaviors rather than being directly linked to favorable metabolic health. Given the cross-sectional nature of this study, causal relationships cannot be established. Future longitudinal studies are needed to determine whether dietary supplement use and overall dietary quality influence metabolic health trajectories during adolescence.

## Figures and Tables

**Table 1:** Baseline characteristics of Korean adolescents according to dietary supplement use.

Characteristics	Supplement Use			*p*-Value
	Total N (%)	No N (%)	Yes N (%)	
Total participants	1550 (100.0)	864 (56.9)	686 (43.1)	
Sex				0.175
Male	799 (51.8)	431 (50.2)	368 (54.0)	
Female	751 (48.2)	433 (49.8)	318 (46.0)	
Age group				<0.001
10–12 years	600 (33.9)	286 (29.1)	314 (40.2)	
13–15 years	557 (34.4)	340 (36.9)	217 (31.0)	
16–18 years	393 (31.7)	238 (33.9)	155 (28.8)	
Household income				0.039
Low	386 (24.3)	229 (26.3)	157 (21.7)	
Lower middle	388 (26.3)	234 (27.6)	154 (24.7)	
Upper middle	383 (24.5)	181 (21.5)	202 (28.6)	
High	387 (24.8)	214 (24.7)	173 (25.0)	
Residence				0.281
Urban	1313 (85.7)	726 (84.8)	587 (86.8)	
Rural	237 (14.3)	138 (15.2)	99 (13.2)	
BMI category				0.002
Underweight	469 (28.5)	226 (24.5)	243 (33.7)	
Normal	676 (44.2)	385 (45.4)	291 (42.6)	
Overweight	168 (11.5)	109 (13.4)	59 (8.9)	
Obese	226 (15.9)	136 (16.7)	90 (14.8)	
Diet quality category				<0.001
Poor	283 (18.3)	193 (22.4)	90(13.1)	
Moderate	1124 (72.6)	606 70.2)	518 (75.5)	
Good	142 (9.2)	64 (7.4)	78 (11.4)	
Metabolic risk clustering				0.356
No	1244 (79.7)	689 (78.7)	555 (80.9)	
Yes	306 (20.3)	175 (21.3)	131 (19.1)	

Note: Supplement use was defined as dietary supplement use during the past year; *p*-values were obtained using Rao–Scott chi-square tests. BMI, Body mass index.

**Table 2:** Multivariate logistic regression analysis for metabolic risk clustering among Korean adolescents.

Variables	Model 1 OR (95% CI)	*p*-Value	Model 2 OR (95% CI)	*p*-Value
Dietary supplement use				
Non-user	1.00		1.00	
Supplement user	0.83 (0.61–1.12)	0.211	0.91 (0.67–1.24)	0.546
Age group				
10–12 years	1.00		1.00	
13–15 years	0.69 (0.50–0.96)	0.026	0.66 (0.47–0.92)	0.016
16–18 years	0.71 (0.49–1.03)	0.072	0.73 (0.51–1.07)	0.103
Sex				
Male	1.00		1.00	
Female	0.54 (0.40–0.74)	<0.001	0.61 (0.44–0.85)	0.003
Household income				
High	1.00		1.00	
Low	1.26 (0.80–2.00)	0.315	1.32 (0.84–2.08)	0.234
Lower middle	0.81 (0.49–1.32)	0.391	0.83 (0.50–1.36)	0.452
Upper middle	1.12 (0.71–1.79)	0.621	1.03 (0.66–1.62)	0.891
Residence				
Rural	1.00		1.00	
Urban	0.91 (0.56–1.47)	0.700	0.87 (0.54–1.41)	0.573
Survey year				
2024	1.00		1.00	
2022	1.23 (0.80–1.87)	0.342	1.25 (0.80–1.94)	0.331
2023	1.19 (0.78–1.82)	0.421	1.18 (0.77–1.81)	0.455
Dietary variables				
Total energy intake (kcal/day)	—	—	1.003 (0.976–1.030)	0.844
Sodium intake (mg/day)	—	—	1.027 (0.961–1.098)	0.431
Carbohydrate percentage (%)	—	—	1.002 (0.987–1.017)	0.776

Model 1: Adjusted for age group, sex, household income, residence, and survey year. Model 2: Additionally adjusted for total energy intake, sodium intake, and carbohydrate percentage.

**Table 3:** Sex-stratified logistic regression analysis for metabolic risk clustering among Korean adolescents.

Variables	Male Adolescents OR (95% CI)	*p*-Value	Female Adolescents OR (95% CI)	*p*-Value
Dietary supplement use				
Non-user	1.00		1.00	
Supplement user	0.72 (0.49–1.05)	0.084	1.10 (0.69–1.75)	0.700
Age group				
10–12 years	1.00		1.00	
13–15 years	1.01 (0.67–1.53)	0.956	0.37 (0.21–0.65)	<0.001
16–18 years	0.90 (0.54–1.49)	0.680	0.53 (0.30–0.91)	0.022
Household income		0.827		0.123
High	1.00		1.00	
Low	1.08 (0.62–1.88)	0.794	1.63 (0.81–3.29)	0.171
Lower middle	0.83 (0.46–1.49)	0.526	0.77 (0.35–1.70)	0.512
Upper middle	0.93 (0.54–1.60)	0.788	1.56 (0.75–3.24)	0.235
Residence				
Rural	1.00		1.00	
Urban	0.79 (0.42–1.47)	0.452	1.09 (0.57–2.08)	0.797
Survey year				
2024	1.00		1.00	
2022	1.60 (0.96–2.65)	0.070	0.79 (0.44–1.42)	0.432
2023	1.27 (0.79–2.04)	0.333	1.12 (0.59–2.13)	0.729

OR, odds ratio; CI, confidence interval.

## Data Availability

The datasets used in this study are publicly available in Korea National Health and Nutritional Examination Survey (KNHANES) data archives: https://knhanes.kdca.go.kr/knhanes/main.do# (accessed on 24 January 2026).

## Supplementary Materials

The following supporting information can be downloaded at: https://www.mdpi.com/article/10.3390/foods15142554/s1, Figure S1. Flowchart for population selection; Table S1. Primary outcome and mediation variables with covariates categories; Table S2. Diet quality scoring system used among Korean adolescents; Table S3. Definition of metabolic risk components; Figure S2. Distribution of metabolic indicators; Table S4. Clinical and nutritional characteristics among Korean adolescents; Table S5. Survey-weighted linear regression analyses of individual metabolic components according to dietary supplement use; Table S6. Survey-weighted logistic regression analysis for metabolic risk clustering according to obesity status; Table S7. Survey-weighted logistic regression Joint test for the interaction between dietary supplement use and sex.
