# Supplementary material for: An Exploratory Indirect Association Analysis of Dietary Supplement Use and Metabolic Risk in Korean Adolescents: A Cross-Sectional Study
Source: Foods. 2026 Jul 20;15(14):2554. doi: 10.3390/foods15142554 (PMC13409285; doi:10.3390/foods15142554)
Supplement: Supplementary file 1 [file foods-15-02554-s001.zip › foods-4406957-supplementary.pdf]

Supplementary materials

Supplementary Figure S1.

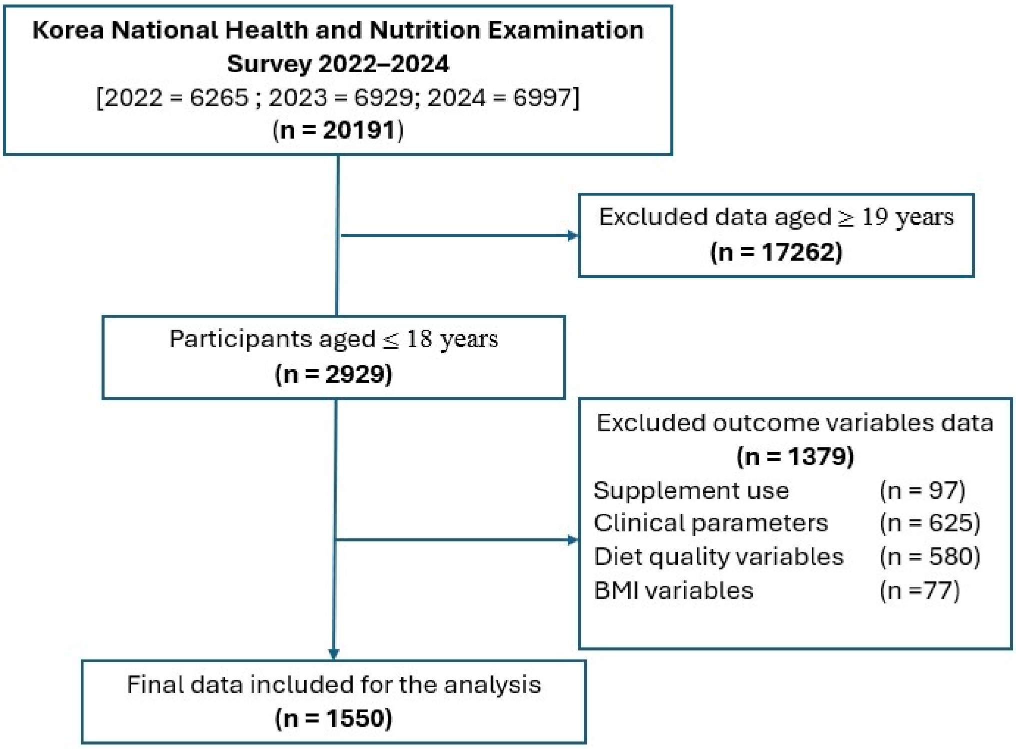

Figure S1. Flowchart for population selection

Table S1. Primary outcome and mediation variables with covariates categories

Domain	Variable	Definition/Measurement	Categories/Scale
Exposure	Dietary supplement use	Assessed using the KNHANES nutrition questionnaire. Participants were classified according to whether they consumed any dietary supplement during the previous 12 months.	Yes / No
Mediator (Exploratory)	Diet quality score	A modified diet quality score based on the Korean Dietary Guidelines (continuous and categories)	Continuous (0–5)
Primary outcome	Metabolic risk clustering	Defined as the presence of ≥2 abnormal metabolic components. Detailed cut-off values are presented in Supplementary Table S2.	Yes / No
Secondary outcomes	Individual metabolic components	Body mass index (BMI, kg/m²), waist circumference (cm), fasting glucose (mg/dL), triglycerides (mg/dL), HDL-cholesterol (mg/dL), and systolic/diastolic blood pressure (mmHg).	Continuous
Covariates	Age group	Age at survey participation	10–12, 13–15, 16–18 years
	Sex	Self-reported sex	Male, Female
	Household income	Household income quartiles provided by KNHANES	Low, Lower-middle, Upper-middle, High
	Residential area	Place of residence	Urban, Rural
	Survey year	KNHANES survey cycle	2022, 2023, 2024

Table S2. Diet quality scoring system used among Korean adolescents

Dietary component	Criteria	Score
Energy intake adequacy	Daily energy intake within recommended range for age and sex	1
	Outside recommended range	0
Carbohydrate proportion	Carbohydrate intake within 55–65% of total energy intake	1
	<55% or >65% of total energy intake	0
Regular breakfast consumption	Consumed breakfast regularly (≥5 days/week)	1
	Irregular breakfast consumption (<5 days/week)	0
Vegetable intake frequency	Daily vegetable consumption	1
	Non-daily vegetable consumption	0
Sodium intake	Sodium intake <2,000 mg/day	1
	Sodium intake ≥2,000 mg/day	0

Diet quality score classification

Total score	Diet quality category
0–2 points	Poor
3–4 points	Moderate
5–6 points	Good

Table S3. Definition of metabolic risk components

Metabolic component	Definition	Cut-off value	Reference
General obesity (BMI)	Age- and sex-specific body mass index	BMI ≥95th percentile for age and sex or BMI ≥25.0 kg/m² (whichever criterion is met)	2017 Korean National Growth Charts; Korean Society of Pediatric Endocrinology
Abdominal obesity (Waist circumference)	Age- and sex-specific waist circumference	Waist circumference ≥90th percentile for age and sex	International Diabetes Federation (IDF, 2007)
Elevated fasting glucose	Fasting plasma glucose	≥100 mg/dL (5.6 mmol/L)	American Diabetes Association (ADA, 2024); IDF
Hypertriglyceridemia	Serum triglycerides	≥150 mg/dL (1.7 mmol/L)	IDF Consensus Definition (2007)
Low HDL-cholesterol	HDL-C concentration	<40 mg/dL (1.03 mmol/L)	IDF Consensus Definition (2007)
Elevated blood pressure	Systolic and/or diastolic blood pressure	SBP ≥130 mmHg and/or DBP ≥85 mmHg	IDF Consensus Definition (2007)
Metabolic risk clustering	Composite outcome	Presence of ≥2 abnormal metabolic components	Modified from IDF Consensus Definition

BMI, body mass index; HDL-C, high-density lipoprotein cholesterol; SBP, systolic blood pressure; DBP, diastolic blood pressure.

Note: Metabolic risk clustering was defined as the presence of two or more abnormal metabolic components

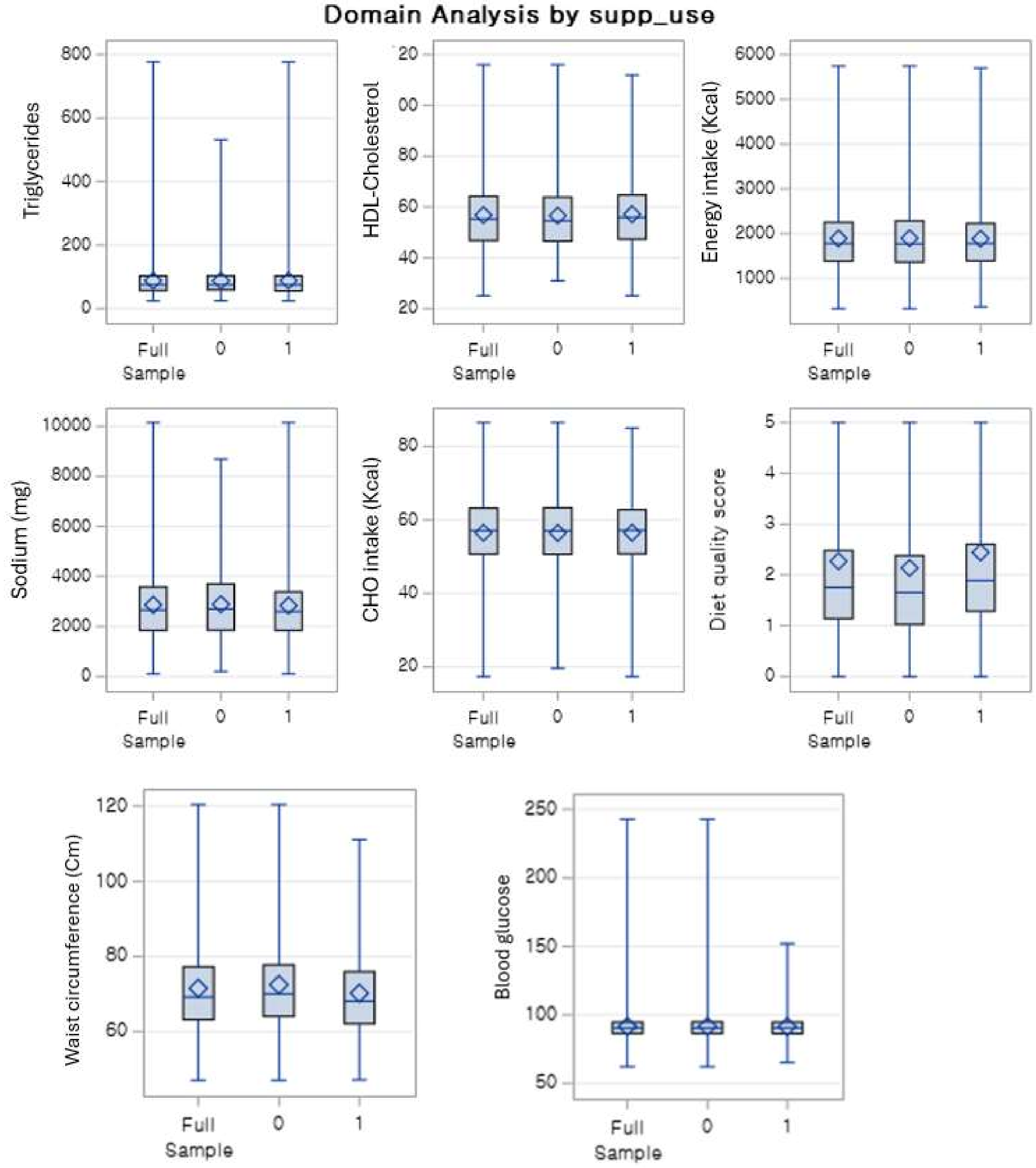

Figure S2. Distribution of metabolic indicators (serum triglycerides, HDL-cholesterol, total energy intake, sodium intake, carbohydrate energy percentage, obesity (WC), blood glucose and diet quality score) according to dietary supplement use among Korean adolescents. Boxplots present the median, interquartile range (IQR), minimum and maximum values, with diamonds indicating the weighted mean. Group 0 represents non-users of dietary supplements, and Group 1 represents dietary supplement users.

Table S4. Clinical and nutritional characteristics among Korean adolescents

Variables	Mean	Standard Error
Clinical parameters		
Body mass index (kg/m²)	21.17	0.14
Waist circumference (cm)	71.52	0.38
Fasting glucose (mg/dL)	91.49	0.28
Triglycerides (mg/dL)	88.10	1.94
HDL-cholesterol (mg/dL)	56.83	0.46
Dietary intake variables		
Total energy intake (kcal/day)	1892.85	22.96
Protein intake (g/day)	72.99	1.07
Carbohydrate intake (g/day)	263.68	3.25
Sodium intake (mg/day)	2862.72	42.31

Table S5. Survey-weighted linear regression analyses of individual metabolic components according to dietary supplement use among Korean adolescents, KNHANES 2022–2024

**Metabolic component variables**	**β (95% CI)[a] for Supplement use vs. non-use**	**p**	**R^2**
Body mass index (kg/m^2)	−0.69 (−1.21, −0.18)	**0.009**	0.131
Waist circumference (cm)	−1.71 (−3.02, −0.41)	**0.010**	0.206
Fasting glucose (mg/dL)	−0.53 (−1.58, 0.53)	0.330	0.071
Triglycerides (mg/dL)	1.27 (−6.34, 8.87)	0.744	0.013
HDL-cholesterol (mg/dL)	0.17 (−1.40, 1.73)	0.834	0.079
Systolic blood pressure (mmHg)	−0.79 (−1.95, 0.37)	0.183	0.133
Diastolic blood pressure (mmHg)	−0.23 (−1.09, 0.63)	0.599	0.048

β, regression coefficient; CI, confidence interval; HDL, high-density lipoprotein.

[a] Models were adjusted for age group, sex, household income, residential area, and survey year.

Table S6. Survey-weighted logistic regression analysis for metabolic risk clustering according to obesity status among Korean adolescents, KNHANES 2022–2024

Variables	aOR	95% CI	p-value
**Obesity status**			**<0.001**
Obese	Reference	—	—
Non-obese	0.21	0.14–0.31	<0.001
**Age group**			**0.001**
10–12 years	Reference	—	—
13–15 years	0.61	0.43–0.87	0.007
16–18 years	0.52	0.36–0.76	<0.001
**Sex**			**0.006**
Male	Reference	—	—
Female	0.65	0.47–0.88	0.006
**Household income**			0.367
High	Reference	—	—
Low	1.23	0.77–1.96	0.389
Lower middle	0.84	0.50–1.39	0.489
Upper middle	1.21	0.75–1.94	0.444
**Residence**			0.700
Rural	Reference	—	—
Urban	1.10	0.68–1.76	0.700
**Survey year**			0.466
2024	Reference	—	—
2023	1.25	0.81–1.94	0.319
2022	1.30	0.83–2.03	0.248

Model statistics: c-statistics = 0.678; Wald test p<0.001.

Table S7. Survey-weighted logistic regression Joint test for the interaction between dietary supplement use and sex

**Interaction Effect**	**F value**	**p-value**
Dietary supplement use	4.03	0.045
Sex	16.12	<0.001
**Dietary supplement use × Sex**	2.82	0.094

Note: Survey-weighted logistic regression adjusted for age group, household income, residential area, and survey year. The interaction between dietary supplement use and sex was not statistically significant (*P* = 0.0938), indicating no evidence of effect modification by sex.
